# Fever-Induced Brugada Sign: Clue for Clinical Management with Non-Negligible Risk of Sudden Cardiac Death

**DOI:** 10.3390/jcm12103503

**Published:** 2023-05-16

**Authors:** Piotr Bijak, Vassil B. Traykov, Avi Sabbag, Sergio Conti, Christian Sohns, Paweł T. Matusik

**Affiliations:** 1 Cardiology Outpatient Clinic, The John Paul II Hospital, 31-202 Kraków, Poland; 2 Department of Invasive Electrophysiology and Cardiac Pacing, Acibadem City Clinic Tokuda Hospital, 1407 Sofia, Bulgaria; 3 The Davidai Center for Rhythm Disturbances and Pacing, Chaim Sheba Medical Center, Tel Hashomer 52621, Israel; 4 Department of Cardiology, Electrophysiology Department, ARNAS Ospedali Civico Di Cristina Benfratelli, 90127 Palermo, Italy; 5 Clinic for Electrophysiology, Herz- und Diabeteszentrum NRW, Georgstr. 11, 32545 Bad Oeynhausen, Germany; 6 Department of Electrocardiology, Institute of Cardiology, Faculty of Medicine, Jagiellonian University Medical College, 31-202 Kraków, Poland; 7 Department of Electrocardiology, The John Paul II Hospital, 31-202 Kraków, Poland

Brugada syndrome (BrS) is a primary electrical disease predisposing to ventricular tachyarrhythmias and sudden cardiac death [1]. The optimal diagnostics and risk stratification in patients suspected of having BrS are challenging [2]. A type 1 Brugada electrocardiogram (ECG) pattern is observed in about 2% of patients with fever and is also described in pediatric inflammatory multisystem syndrome related to COVID-19 [3,4]. The current European Society of Cardiology (ESC) guidelines for the management of patients with ventricular arrhythmias and the prevention of sudden cardiac death indicate the necessity to exclude Brugada phenocopies while making a diagnosis of BrS [5]. These guidelines highlight the lower specificity of the type 1 Brugada ECG pattern observed during the sodium channel blocker test or fever [5], but it should be mentioned that these induced Brugada ECG patterns are not considered a BrS phenocopy. In these conditions, genetic testing may be considered, according to a recent expert consensus statement on the state of genetic testing for cardiac diseases [6].

In the Journal of Clinical Medicine Special Issue “New Frontiers in Electrocardiography, Cardiac Arrhythmias, and Arrhythmogenic Disorders”, Tsai et al. [7] described a long-term follow-up of a cohort of patients with a fever-induced type 1 Brugada ECG pattern. They included 18 asymptomatic patients without a spontaneous type 1 BrS ECG pattern and no family history of sudden death and 3 symptomatic individuals (two had previous syncopal episodes, while one had aborted sudden cardiac arrest). In the mean follow-up almost 10 years later, none of the asymptomatic patients experienced ventricular arrhythmic events. At the same time, all symptomatic patients experienced cardiac events, including implantable cardioverter-defibrillator therapies (shocks for new arrhythmic events) or sudden death.

Notably, Mizusawa et al. [8] reported the risk of arrhythmic events in patients with fever-induced BrS type 1 ECGs in a relatively large, multicenter, retrospectively collected cohort. In their cohort, the arrhythmic event rate was 3.0%/year in patients with a history of ventricular fibrillation, 1.3%/year in patients with a history of syncope, and 0.9%/year in asymptomatic patients. These results, while possibly derived from a more selected population, emphasize that the risk associated with fever-induced type 1 Brugada ECG is not negligible (compared to the incidence of sudden cardiac death in the general population of 0.03–0.1%/year [9]).

Moreover, in their paper, Tsai et al. [7] noted that there was an increase in heart rate and a significant shortening of the PR interval during periods of fever. In contrast, QRS duration and QTc intervals were not different during fever, compared to ECGs recorded without this condition in both asymptomatic and symptomatic patients. These data are consistent with the observations of Mizusawa et al. [8]. Interestingly, Mizusawa et al. [8] additionally observed that PR interval, QRS duration, and QTc interval prolonged during the sodium channel blocker challenge, suggesting different mechanisms implicated in their induction.

The association between fever and ECG pattern may be particularly important in light of evidence for inflammation in the right ventricular outflow tract of BrS patients, which may trigger ventricular arrhythmias in predisposed hearts [10].

It should be mentioned that a fever-induced type 1 Brugada ECG pattern may be awarded 3 points in the proposed Modified Shanghai scoring system for the diagnosis of BrS, compared to 3.5 points for a spontaneous type 1 Brugada ECG pattern and 2 points for a sodium channel blocker-induced Brugada type 1 ECG pattern (all at nominal or high leads) [6]. Moreover, the panel of experts for the recent ESC guidelines recommends that an induced type 1 ECG pattern requires other clinical characteristics, including arrhythmic syncope, polymorphic ventricular tachycardia or ventricular fibrillation, and consistent family history to diagnose BrS, contrary to spontaneous type 1 Brugada ECG pattern in patients without other heart diseases [5].

Thus, in line with current ESC guidelines [5], we consider a close follow-up with general recommendations (among others: avoidance of drugs and other substances listed on http://www.brugadadrugs.org (accessed on 24 April 2023) and treatment of fever with antipyretic drugs) of asymptomatic (no arrhythmic syncope/nocturnal agonal respiration) patients with fever-induced type 1 Brugada ECG pattern without documented ventricular arrhythmia and with negative family history (both of BrS and sudden death < 45 years) as the most reasonable approach to clinical management.
